# RNA-Seq Analysis of Ruminal Methane Emissions in Beef-on-Dairy Cattle: Evidence for Immune, Nervous, and Endocrine Pathway Involvement

**DOI:** 10.3390/ani16040589

**Published:** 2026-02-13

**Authors:** Vahid Razban, Omar Cristobal Carballo, Steven Morrison, Masoud Shirali

**Affiliations:** Agri Food and Bioscience Institute, AFBI Hillsborough Large Park Hillsborough, Belfast BT26 6DR, UK; vahid.razban@afbini.gov.uk (V.R.); omarcristobal.carballo@afbini.gov.uk (O.C.C.); steven.morrison@afbini.gov.uk (S.M.)

**Keywords:** ruminal methane, beef cattle, transcriptomics, biomarker, gene enrichment, cell type enrichment

## Abstract

Methane (CH_4_) emissions present a significant challenge to both environmental sustainability and energy efficiency in ruminants, including beef cattle that are born in dairy herds. Although numerous approaches, including alterations in feed and the use of additives, are under investigation to mitigate these emissions, the genetic selection of animals that produce lower levels of methane offers the potential for enduring and cumulative advantages. Transcriptome analysis represents a crucial advancement in elucidating the networks and mechanisms through which the ruminant genome influences methane emissions. In the present study, methane emissions were measured using a GreenFeed system in beef-on-dairy cattle (*n* = 11). High-throughput RNA sequencing was conducted on animal blood samples, followed by differential gene expression analysis using methane production (g/d) as a continuous trait. The analysis identified eleven differentially expressed genes (DEGs), including six downregulated (*KIAA1211L*, *LOC107131224*, *OSCP1*, *IL12B*, *LOC618859*, *FREM1*) and five upregulated (*DSCAML1*, *OSBP2*, *ACAN*, *PRSS16*, *CD1B*) genes (*P*adj < 0.05) with one gene exhibiting potential biomarker characteristics. Gene and cell enrichment, as well as pathway analysis, suggested that nervous, immune, and endocrine systems may be involved in ruminal methane production by beef-on-dairy cattle. These findings highlight the potential of transcriptomic biomarkers to guide genetic selection strategies, offering a sustainable pathway to reduce methane emissions and enhance both environmental and agricultural efficiency.

## Simple Summary

Methane produced and emitted by cattle is a major contributor to climate change and represents a loss of dietary energy in livestock systems. While dietary interventions can help reduce these emissions, selecting animals that naturally produce less methane may offer long-term benefits. In this study, we measured methane emissions from a group of beef cattle born to dairy cows and collected blood samples to study their genes. Using advanced genetic techniques, we identified 11 genes associated with individual methane production. Further analysis suggested that the nervous system, immune system, and hormone-related processes might play a role in how much methane cattle produce. These findings may support breeding strategies that reduce methane emissions and contribute to more environmentally sustainable production.

## 1. Introduction

Climate change driven by greenhouse gas (GHG) emissions is a major global concern, highlighting the need to reduce GHGs such as enteric methane (CH_4_) from ruminants [1,2]. Methane, a byproduct of the ruminant digestive process, is a GHG 28 times more potent than carbon dioxide and accounts for 2–12% of energy loss in livestock [2,3,4]. Energy loss through ruminal methane production has been estimated at around 6%, indicating that methane mitigation can reduce the carbon footprint of the ruminant sector while improving nutrient utilization and production efficiency [5].

Therefore, it is imperative to identify strategies for mitigating methane emissions, a byproduct of the ruminant digestive process, which is essential not only for diminishing energy losses in livestock but also for enhancing productivity and addressing the global greenhouse gas effect. In addition to dietary interventions and feed additives, gene-editing strategies targeting forage crops and rumen methanogens also represent means of mitigating ruminal methane production. The heritability of methane production has been reported to range from 0.12 to 0.52, which indicates that selective breeding could be utilized as a durable approach to achieving this aim [1,3,6].

Methane emissions resulting from enteric fermentation is a hard-to-measure phenotype and measuring technologies, including respiration chambers, Sulfur Hexafluoride (SF6) tracers, and infrared gas analysis techniques such as GreenFeed Systems, are expensive and not widely available [7]. Under practical conditions, this makes the implementation of genetic selection for reduced methane production and improvement of herd genetic merit challenging [8]. For difficult-to-measure traits such as hoof lesions and mastitis in cattle, genomic selection has been successfully studied and thus could be implemented to reduce ruminal methane production, as well [9,10].

Due to the high cost and the time- and labour- intensive nature of methane measurement, marker-assisted selection (MAS) or genomic selection offer alternative approaches that leverage predictive DNA, RNA, protein, and metabolite markers. These methods aim to overcome the limitations of conventional genetic selection, which relies heavily on phenotypic data [5]. The discovery of biomarkers, including molecular markers associated with difficult-to-measure traits, could provide a cost-effective and rapid tool for predicting methane production in ruminants, as has been recently reported for residual feed intake in lactating Holsteins [11]. In this context, elucidation of the underlying biology is essential for achieving genetic progress and understanding the roles and associations of genes and proteins with ruminal methane production. Another consideration is that a comprehensive understanding of the genetic background of methane production is crucial as effective methane mitigation must be achieved without negatively impacting economic traits [12].

Despite the role of ruminant genotypes on methane emission, it is still largely unknown how genetics exerts its effects and what the underlying mechanisms are [1,13]. Transcriptome analysis represents a crucial advancement in elucidating the networks and mechanisms through which the ruminant genome influences methane emissions.

The dairy-cattle population significantly contributes to the overall beef production in numerous countries, frequently exceeding the output of the local beef-cattle population [14]. It is probable that the disparity in contributions to national beef production from dairy and beef herds will continue to increase in the future, particularly as the profitability gap between dairy and beef operations expands in many regions. While beef is commonly regarded as a secondary product of the dairy industry, it serves as an important source of revenue for dairy farms and constitutes a substantial and increasing share of beef production in various countries [14]. The aim of this study was to investigate the transcriptomic profiles of beef-on-dairy cattle to identify genes and biological pathways significantly associated with ruminal methane emissions. By integrating methane measurements with RNA sequencing data, we aimed to uncover potential candidate biomarkers that could facilitate marker-assisted or genomic selection for reduced methane production.

## 2. Materials and Methods

### 2.1. Animals, Methane Measurement, and Sample Collection

Twelve Holstein × Angus crossbred castrated males from a commercial rearing setting at Agri-Food Biosciences Institute, Loughgall, were selected for this study. Steers had an average body weight of 605 kg and an average age of 22 months. Animals were reared in a slated floor barn with access to automatic feeders. A fattening diet was provided for 50 and 100 days until animals reached a desired carcass weight of 280 kg. The diet consisted of good-quality grass silage with ad libitum access and 5 kg of commercial concentrate. One kg of the total five kg concentrate was provided by a GreenFeed unit. Methane measurements (gram CH_4_ per day) were carried out using a GreenFeed unit (C-Lock Inc., Rapid City, SD, USA). The feeding schedule was as follows: 6 visits a day, 6 drops per visit, and 35 s between drops. Animals were allowed one visit to the unit every four hours, for a total of six visits per day. To improve the quality of data, methane measurements were performed for approximately 50 days and animals with less than 30 measurement days or fewer than 50 valid visits during this period were removed from the analysis. Blood samples from the animals were collected at the time of slaughter, after completing methane measurements. On the final day, these samples were placed into PAXgene Blood RNA tubes (BD, Franklin Lakes, NJ, USA; and QIAGEN, Venlo, The Netherlands) and stored at −80 °C until analysis. No ethical statements were required for this process, as the samples were obtained post-slaughter rather than from live animals.

### 2.2. RNA Sequencing and Pre-Processing of Raw Read Data

Blood samples were used for high-throughput RNA sequencing on the Illumina NovaSeq platform (Illumina, San Diego, CA, USA). Sequencing weas performed using NovaSeq instrument control software (bcl2fastq2, v2.20.0). The paired-end sequencing was performed with 30 million reads coverage per sample and 150 bp length per read.

The paired-end RNA-seq data with 30 million reads coverage were received and underwent pre-processing including quality control (FastQC, V0.11.9) [15] and trimming (Trimmomatic, 0.39) [16] cycles to provide high-quality read sequences for alignment on the reference genome. Through these cycles, the adapter sequences and low-quality sequences were removed. The QC of RNA-seq data in pre-processing included assessment of read quality scores, adapter contamination, GC content, sequence duplication, and overall base composition.

### 2.3. Gene Mapping and Generation of the Read Count Matrix

*Bos taurus* reference genome ARS-UCD1.2 [17] was used to align the final trimmed read sequences using STAR 2.7.11b [18]. The aligned sequences were indexed using Samtools 1.10 [19], and the read count matrix was generated by FeatureCounts, 2.0.2 [20]. To reduce false positives, a read pair was counted only when both forward and reverse reads aligned to the same gene. This approach improves specificity in gene assignment and reduces potential miscounts from discordant or ambiguous mappings. The count matrix was used for differential gene expression analysis with DESeq2 1.46.0 and for functional analysis with iDEP 2.0. DESeq2 differential gene expression analysis was performed to identify genes whose expression levels vary significantly with methane production. The iDEP offers an integrated platform for gene set enrichment and pathway exploration to perform functional analysis.

### 2.4. Differential Gene Expression

The gene count matrix, including estimated expression per gene, was used as input for differential gene expression analysis to detect differentially expressed genes (DEGs) for average methane production per animal, as a continuous trait, by DESeq2 package [21] in R (v 4.4.0). Average methane production for each animal was included in the DESeq2 model and significant DEGs were identified using *P*adj < 0.05. Statistical analysis revealed no confounding variables; therefore, the DESeq2 model was fitted with methane records included. Differential gene expression analysis by DESeq2 uses a generalized linear model in which methane production is treated as a numeric covariate in the design formula and resulting *p*-values are then adjusted for multiple testing.

### 2.5. Dimensionality Reduction Analyses

Dimensionality reduction analyses were performed to explore sample structure and potential hidden variation. Principal Component Analysis (PCA) was conducted using variance-stabilized expression data generated by the DESeq2 package in R. Uniform Manifold Approximation and Projection (UMAP) was performed using the umap package [22] (0.2.10.0) to visualize non-linear relationships among animals.

### 2.6. Sequence Alignment

Nucleotide BLAST v2.17.0 was used to identify sequences with high similarity to the query sequences, and the resulting hits were subsequently aligned using Clustal Omega v1.2.4 [23] to generate a multiple sequence alignment.

### 2.7. Functional Enrichment Analysis

iDEP 2.0 and iDEP 0.96 [24] versions were used to explore gene expressions, phenotype relationships, and pathway enrichment analysis. As part of data pre-processing, genes that were not expressed in any library (sample), as well as those with extremely low expression, were filtered out by iDEP. By default, a gene must have more than 0.5 counts per million (CPM) in at least one sample (n libraries = 1) to be kept in the analysis. The data normalization was set to be performed by the CPM function in edgeR and only genes with levels above min CPM in at least n libraries were retained.

In the context of enrichment analysis conducted via iDEP, *p*-values are derived from a one-sided hypergeometric test, which subsequently undergoes adjustment for multiple comparisons through the Benjamini–Hochberg method, resulting in the calculation of the false discovery rate (FDR). The background gene set comprises filtered genes selected from the initial gene list, specifically those that have met a low threshold in RNA sequencing data. The significant pathways are then ranked based on their FDR values, with only the top 10 pathways being presented.

Tissue, cells, and gene associations from PanglaoDB Augmented 2021 [25] within the Enrichr platform were used to identify the tissues and cell types associated with the DEGs. Because Enrichr libraries are primarily based on human gene annotations, DEGs without human matches were excluded. Bovine genes whose symbols were identical to their human orthologs were retained for downstream analysis. Given the strong conservation of biological pathways across mammals, this comparative strategy enabled us to extract biologically meaningful insights into pathways potentially related to methane production, even in the absence of cattle-specific enrichment libraries.

### 2.8. Statistical Analysis

Genes with *p*-adjusted (*P*adj) < 0.05, regardless of log2 fold change, were considered as DEGs by DESeq2. Regression analysis using R programming (v 4.4.0) was performed to investigate the relationship between the gene expression levels of DEGs and CH_4_ production in sampled animals. The effects of animal breed, age, and feed intake were investigated using a regression model and R programming (*p*-value < 0.05). Using R programming and the DESeq2 package, raw counts for DEGs were extracted from the count matrix and normalized by variance stabilizing transformation (VST). The normalized counts were used to plot a heatmap for DEGs against methane measurements. Using VST-normalized expression for DEGs the slope (β), 95% CI, *p*-value, adjusted R^2^, and RMSE were calculated. Model assumptions were assessed via Shapiro–Wilk (normality), Breusch–Pagan (homoscedasticity), and RESET (linearity), with influence diagnostics (Cook’s distance, leverage, studentized residuals) and leave-one-out (LOOCV) refits to quantify slope stability. Heteroscedasticity-consistent (HC3) standard errors were computed for robust inference. Robustness was evaluated via non-parametric pairs bootstrap (R = 2000), reporting the bootstrap median β, and 95% confidence intervals. To assess bias introduced by cohort homogeneity, unadjusted methane slopes were compared with covariate-adjusted models including age, feed intake, and breed. Following the completion of the analysis using iDEP, pathways were initially filtered according to a FDR threshold of 0.05.

## 3. Results

Due to the insufficient number of methane measurements for one animal, it was removed from our analysis. Further analysis was conducted on the 11 animals that met the criteria outlined in the Section 2. The methane measurements ranged from 233.52 to 332.16 g per day, with standard deviation of 25.85.

Detailed information on sequencing, mapping and the read count matrix is provided in Tables S12 and S13 of the Supplementary Materials. The total number of paired-end reads, each with 150 base pairs, for 11 animals was 554,600,000, of which 64.9% to 71.2% mapped efficiently to the reference genome after QC and trimming. This modest variation reflects expected differences in transcriptome composition, repetitive and paralogous regions, and unannotated or polymorphic loci that reduce confident alignment. All libraries showed uniform sequencing quality, and downstream quantification used stringent featureCounts settings to ensure high-precision gene-level assignment. A total of 20,959 genes were detected by successfully mapping to annotated gene features in the ARS-UCD1.2 genome assembly.

**Figure 1:**
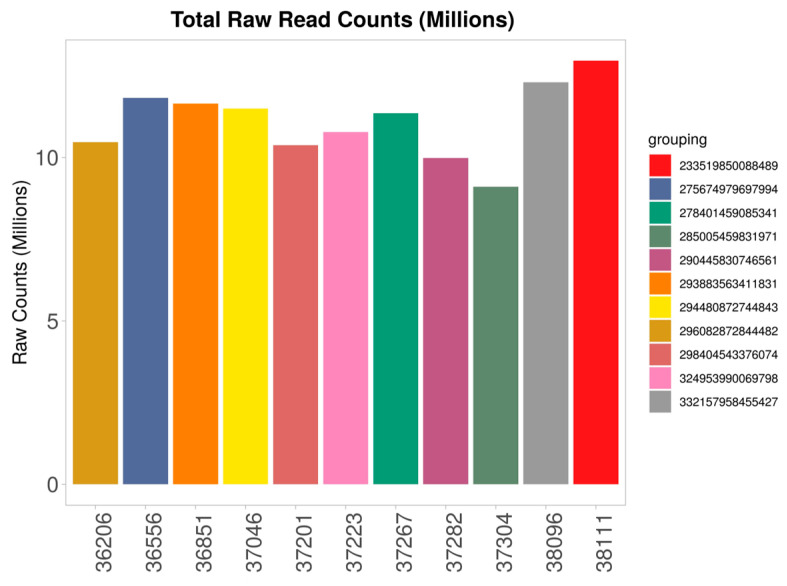
Total raw reads per sample using iDEP 2.0. The average number of methane measurements per animal (Gram/Day) are presented in descending order by different colours in the right panel.

Using iDEP pre-processing, the total raw reads per sample were extracted (Figure 1). Of the total number of genes, 15,181 passed the filter, 12,980 genes were converted to Ensembl gene IDs, and 2201 genes were kept in the data using original IDs.

Exploratory analyses using PCA and UMAP revealed no distinct clustering among the 11 animals, indicating the absence of strong batch effects or biological subgroups (Supplementary Materials Figure S1). We used regression models to evaluate the influence of animals-level covariates, including breed composition, age, and feed intake over a 35-day period, on methane production. None of these covariates were statistically significant (*p* > 0.05) and therefore were excluded from the DESeq2 model. This decision reflects a parsimonious modelling approach aimed at preserving statistical power and avoiding overfitting, particularly given the limited sample size (*n* = 11). Details of these analyses are provided in Supplementary Materials Figure S2, Tables S1–S3.

Differential gene expression analysis using DESeq2 identified six significantly downregulated (*KIAA1211L*, *LOC107131224*, *OSCP1*, *IL12B*, *LOC618859*, *FREM1*) and five upregulated genes (*DSCAML1*, *OSBP2*, *ACAN*, *PRSS16*, *CD1B*) (*p*adj < 0.05) (Table 1).

**Figure 2:**
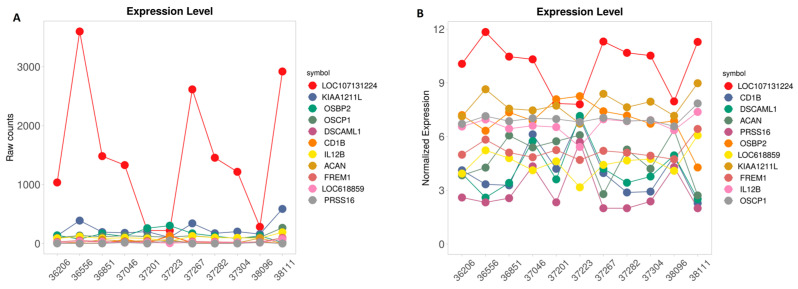
Raw (**A**) and normalized (**B**) expression levels of DEGs for animals that were extracted from count matrix by iDEP.

The raw and normalized expression levels of the above DEGs were extracted using iDEP and illustrated in Figure 2A and Figure 2B, respectively.

**Figure 3:**
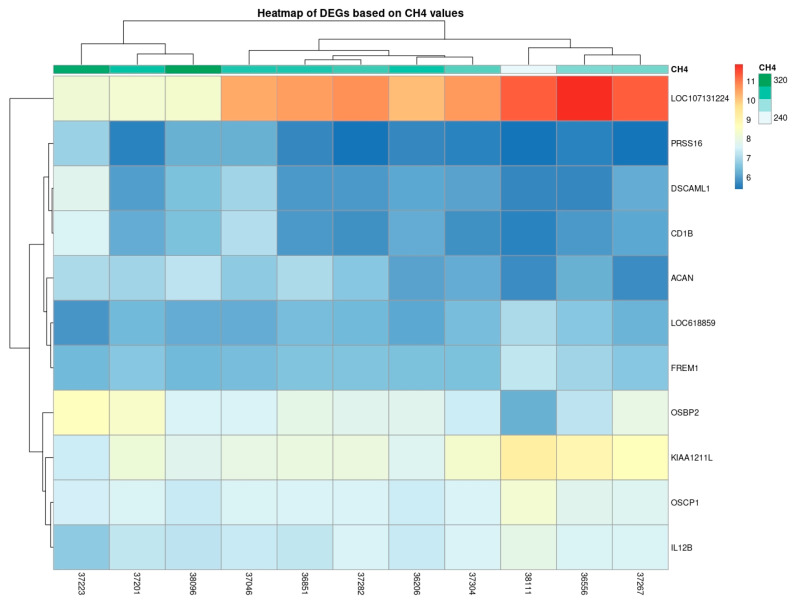
Heatmap of normalized DEGs based on methane (CH_4_) measurements.

The normalized counts were used to create a heatmap based on methane (CH_4_) measurements for each animal (Figure 3). The heatmap was used to visually explore associations between methane levels and expression of DEGs.

*LOC107131224* was clustered separately, suggesting a distinct expression pattern across animals. This may reflect different regulatory mechanisms or unique biological roles in methane production.

As is shown in the vertical dendrogram (Figure 3), *LOC107131224* clustered exclusively from the other DEGs. The other DEGs sub-clustered in two groups with up- and downregulation in the blood samples of animals with different methane productions. On the horizontal dendrogram, the animals sub-clustered into three high, medium, and low methane production, corresponding to the low, medium, and high level of *LOC107131224* expression, respectively.

**Figure 4:**
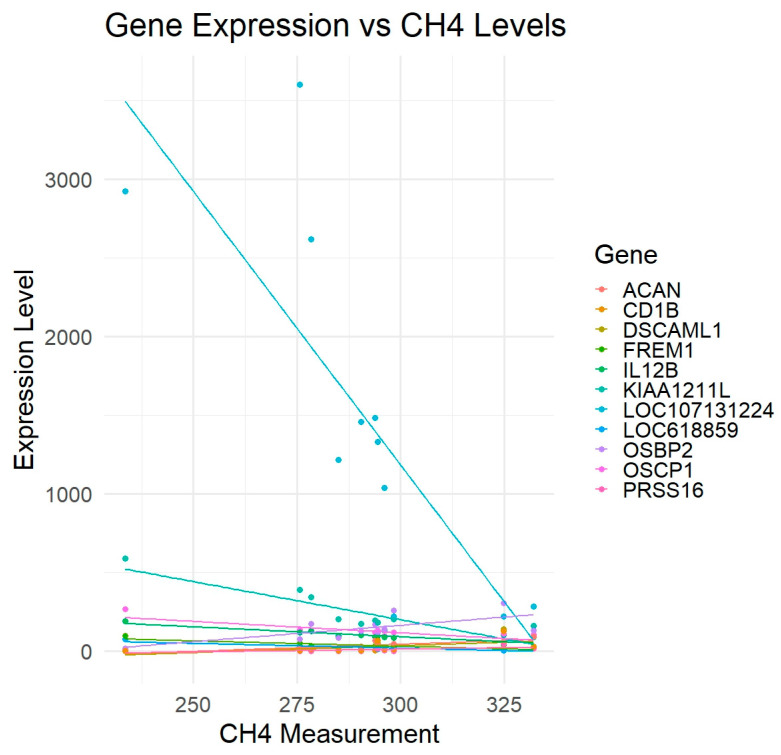
Regression of DEGs expression and methane measurements (g/day).

To confirm associations, particularly for *LOC107131224*, regression analysis was performed between DEGs’ expression and methane values. Direction and strength of correlation with methane, response of DEGs to the methane production (sensitivity), has been illustrated by a regression plot (Figure 4). The steep slope of *LOC107131224* could indicate stronger sensitivity of *LOC107131224* expression to changes in methane levels, suggesting a potential role as a key driver or biomarker.

Other DEGs exhibited associations but clustered together in the heatmap and showed weaker relationships with methane variation, as indicated by smaller regression coefficients. These suggest the possible co-regulation or shared pathway. Their individual contributions to methane variation are less distinct than *LOC107131224*.

**Figure 5:**
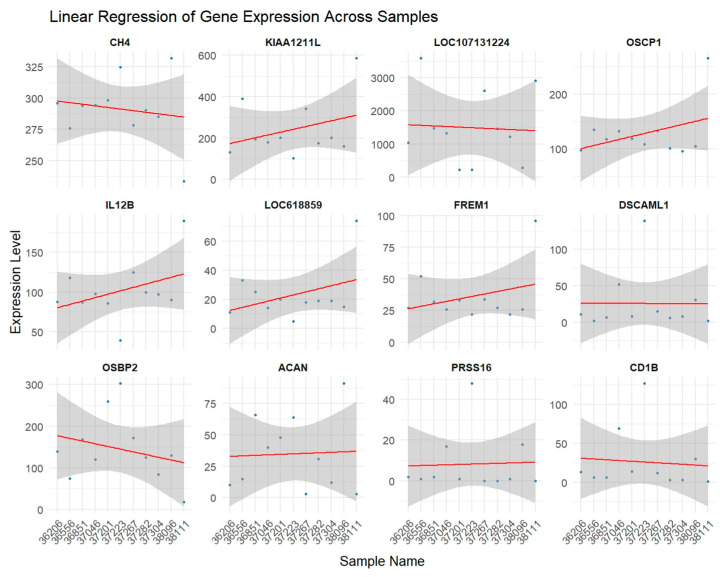
Variations in methane production and gene expression level of DEGs among the animals. The grey area surrounding the red fitted regression line represents the standard error.

Shown in Figure 5 are the results of the regression analysis for methane production and expression of the DEGs among animals. Note that positive or negative associations of the DEGs with methane production across the animals are represented by the fitted regression line in each graph. Estimates of regression parameters presented in Table 2 indicate that *KIAA1211L* explains more variability in methane levels (0.78) than *LOC107131224*, which explains 0.60 of the variability among the animals.

While *LOC107131224* may not provide the best overall fit, the results illustrated in Figure 3 and Figure 4 suggest that it could serve as a valuable marker, exhibiting the most responsiveness to fluctuations in methane levels.

Residual, QQ, scale–location, and leverage diagnostics were comparable across all DEG models. Representative panels for *LOC107131224* are shown in Supplementary Figure S5. Collectively, the panels demonstrate that model assumptions were adequately met and that the differential expression results for DEGs are not materially biassed by residual structure or influential observations. Supplementary Tables S8–S11 summarize the complementary statistical evaluations conducted for all DEGs. Regression confidence intervals, assumption tests, and influence diagnostics collectively supported the adequacy of the fitted models and demonstrated that outlier- or high-leverage points did not alter the inferred methane effects. HC3 robust inference and bootstrap resampling produced consistent slope estimates, reinforcing the robustness of the associations despite the modest sample size. Covariate adjustment revealed only small Δβ values and no changes in effect direction, indicating that age, feed intake, and breed did not materially confound the methane–expression relationships.

Retrieval of the transcript sequence from Ensembl and nucleotide BLAST against human sequences identified *SIGLEC14* and *SIGLEC5* mRNAs (Table 2). The zoomed-out overview of multiple sequence alignment for *LOC107131224* cDNA (transcript: ENSBTAT00000026431.7) against *SIGLEC14* and three isoforms of *SIGLEC5* is presented in Supplementary Materials (Figure S3), highlighting that while the transcripts are truncated, they exhibit partial sequence conservation at specific regions. This visualization was intended to illustrate general alignment patterns rather than detailed residue-level homology. The guide and phylogenetic tree have been provided in Supplementary Materials (Figure S4). *LOC107131224* shows homology to *SIGLEC5/14* family members; further annotation and functional validation are needed.

**Figure 6:**
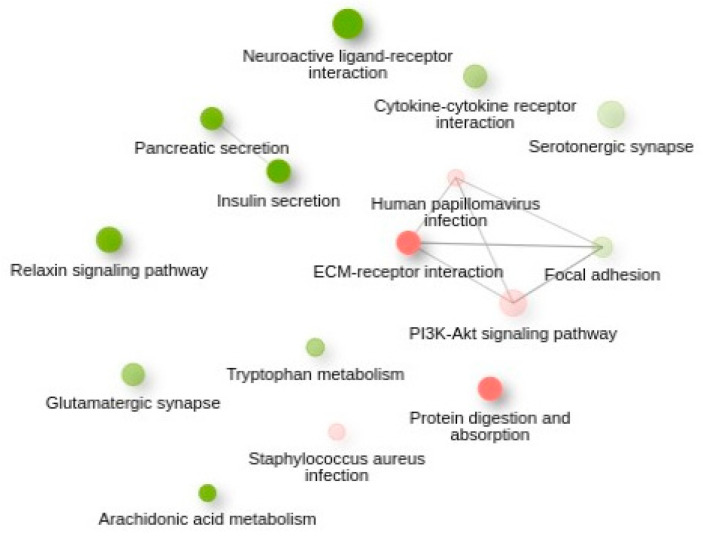
KEGG-enriched pathways analysis based on k-means clustering. In the presented networks, two pathways (nodes) are connected if they share 30% (default, adjustable) or more genes. Green and red represent up- and downregulated pathways, respectively. Darker nodes are more significantly enriched gene sets. Bigger nodes represent larger gene sets. Thicker edges represent more overlapped genes.

The count matrix was used as input for iDEP tools. KEGG (Kyoto Encyclopedia of Genes and Genomes), a database that is widely used to map genes to curated biological pathways, enables interpretation of high-throughput data in the context of cellular and organismal processes. The KEGG was employed for pathway enrichment analysis and the results based on k-means clustering with 2000 top genes and two clusters in *Bos taurus* species revealed the top 10 significantly enriched pathways with lowest FDR (adjusted *p*-value) (Table S4 in Supplementary Materials and Figure 6).

**Figure 7:**
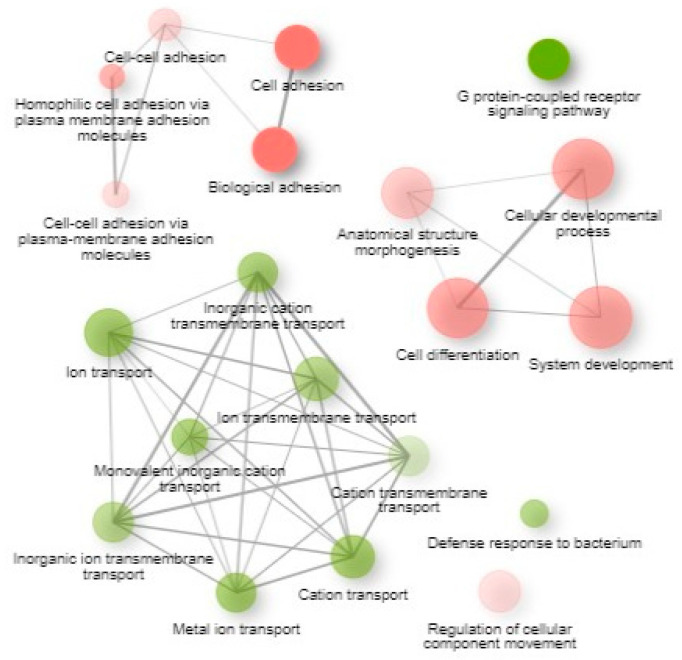
GO biological process enriched pathways based on k-means clustering. In the presented networks, 2 pathways (nodes) are connected if they share 30% (default, adjustable) or more genes. Green and red represent up- and downregulated pathways, respectively. Darker nodes are more significantly enriched gene sets. Bigger nodes represent larger gene sets. Thicker edges represent more overlapped genes.

Results from GO biological process enriched pathways based on k-means clustering (top 2000 genes; 2 clusters) demonstrated significantly enriched biological processes associated with the DEGs (Table S5 in Supplementary Materials and Figure 7). Gene Ontology is a structured vocabulary that categorizes gene functions into biological processes, molecular functions, and cellular components.

**Figure 8:**
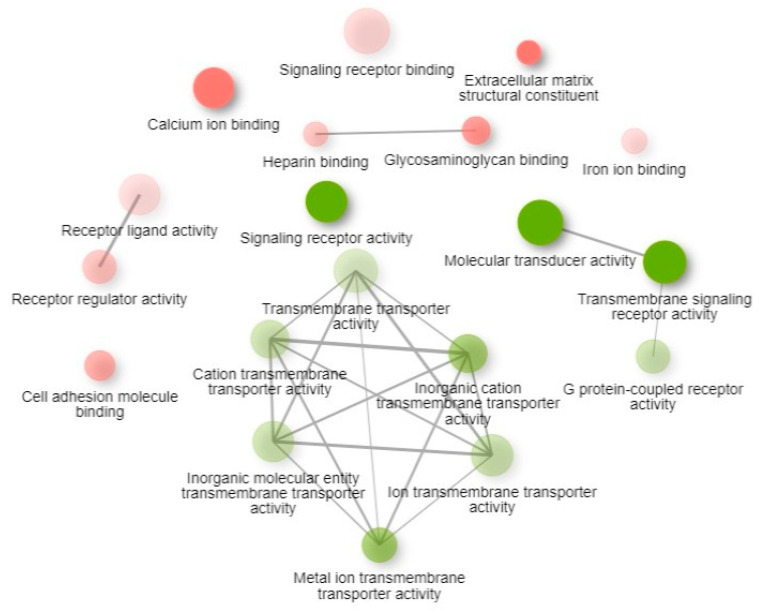
GO molecular function enriched pathways based on k-means clustering. Two pathways (nodes) are connected if they share 30% (default, adjustable) or more genes. Green and red represent up- and downregulated pathways, respectively. Darker nodes are more significantly enriched gene sets. Bigger nodes represent larger gene sets. Thicker edges represent more overlapped genes.

GO molecular function enrichment of the two k-means clusters generated from the top 2000 genes identified the molecular functions significantly associated with the DEGs (Table S6 in Supplementary Materials and Figure 8).

**Figure 9:**
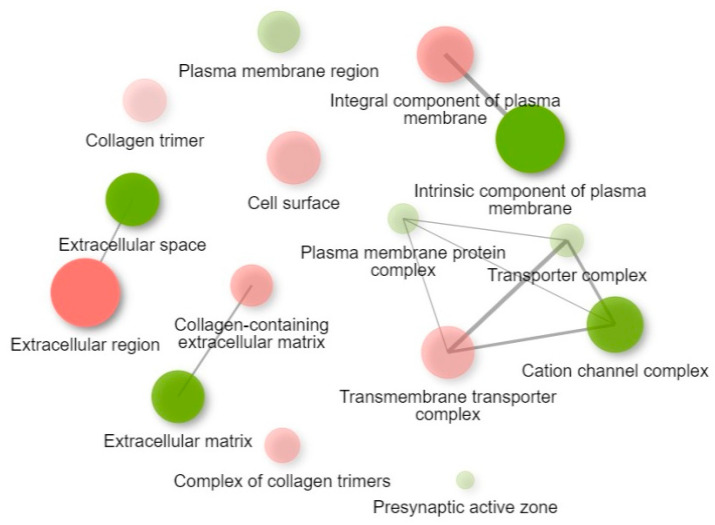
GO cellular component enriched pathways based on k-means clustering. Two pathways (nodes) are connected if they share 30% (default, adjustable) or more genes. Green and red represent up- and downregulated pathways, respectively. Darker nodes are more significantly enriched gene sets. Bigger nodes represent larger gene sets. Thicker edges represent more overlapped genes.

GO cellular component enrichment of the two k-means clusters derived from the top 2000 genes identified the cellular components significantly associated with the DEGs (Table S7 in Supplementary Materials and Figure 9).

The results of enrichment for cell types in PanglaoDB Augmented 2021 are represented in Table 3.

## 4. Discussion

In this study, we identified 11 DEGs exhibiting statistically significant associations with methane emissions (*P*adj < 0.05). Recognizing the inherent limitation of the available sample size, we first established a robustness hierarchy of the detected associations through supplementary analyses of regression confidence intervals, residual distributions, and the potential influence of extreme methane measurements. Additional assessments, including heteroscedasticity-consistent inference, influence diagnostics, and non-parametric bootstrap resampling, collectively supported the stability of the regression models and confirmed that the observed methane–expression relationships were not artefacts of model misspecification or sample homogeneity.

This study sought to elucidate, the genetic, molecular, and cellular mechanisms underlying methane emission in dairy beef cattle, as the host for fermenting microbiome responsible for methane production in the rumen. Utilizing high-throughput RNA sequencing and RNA-seq data analysis revealed the intricate essence of ruminal methane production, encompassing multiple biological systems, pathways, genes, and cellular interactions. A detailed examination of the findings presents intriguing insights that may be valuable for both future applied- and fundamental research endeavours.

Based on the expression variation in DEGs according to the CH_4_ measurements (Figure 2), *KIAA1211L* has the lowest adjusted *p*-value (Table 1). However, the LOC107131224 intriguingly displayed the most pronounced negative association with methane emission across the full measurement range, exhibiting the steepest regression slope (Figure 2 and Figure 3). The substantial variation in *LOC107131224* expression, together with its strong association with methane levels, suggests that this gene may be linked to variation in methane production. Although correlation does not imply causation, this degree of sensitivity indicates that even small differences in methane output correspond to marked differences in LOC107131224 expression, consistent with a potential biomarker role. Given the limited sample size and the incomplete functional annotation of LOC107131224, further validation using qPCR and independent datasets is essential.

Although *IL12B*, *KIAA1211L*, and *LOC618859* showed the lowest *p*-values and the highest R^2^ value (0.7), the association of *LOC107131224* with methane levels, together with its significance in the regression analysis (*p*-value = 0.003; adjusted R^2^ = 0.6), indicates that it may serve as a potential molecular marker for methane production.

Its response to methane fluctuations makes it particularly useful for applications where monitoring and adjusting methane emissions are critical. Thus, *LOC107131224* stands out as a promising candidate for further investigation as a molecular marker for methane production in beef cattle; however, it requires validation with larger samples in future investigations.

The Ensembl database has noted *LOC107131224* (transcript: ENSBTAT00000026431.7) as a novel gene in cattle with 177 orthologues and 16 paralogues, among them the highest matching sequences to the known genes belonging to the *SIGLEC5* and *SIGLEC14* in humans and other species with around 55% sequence match. SIGLECs are a family of sialic-acid-binding immunoglobulin-like lectins and are mostly expressed by immune system cells and are involved in cell–cell interaction and modulate innate and adaptive immune responses [37].

As shown by the multiple sequence alignment in Figure 5, *SIGLEC-5* transcripts seemed to be truncated forms of *SIGLEC-14* mRNA. These highly related SIGLECs are considered paired receptors [37]. SIGLECs are categorized into two subgroups: CD33-related SIGLECs, with around 50–99% sequence identity, and other SIGLECs including *SIGLEC-1*, *SIGLEC-2*, *SIGLEC-4*, and *SIGLEC-15* subgroups, with around 25–30% sequence identity [38].

The GenBank database discontinued *LOC107131224* Gene ID (*SIGLEC14*) in its 4 October 2023, update and subsequently replaced it with *LOC100300478* (*SIGLEC5*) on 30 January 2025. In this update, *SGLEC5* and *SIGLEC14* were presented as the transcript and protein isoforms from the same region of chromosome 18. The SIGLECs are involved in discrimination between self and non-self by the immune system via recognition of sialic acid-containing glycans as ligands on all mammalian cell surfaces. Depending on the involved SIGLECs, they regulate activator and inhibitory receptors on immune cells, which provides the opportunity for sialylated pathogens to be distinguished by the host with or without eliciting immune response [37,38,39]. Most SIGLECs carry immunoreceptor tyrosine-based inhibitory domains on their intracellular motifs. By binding the immune inhibitory SIGLECs to complementary sialoglycans, the immune response is suppressed [40].

Given the indispensable role of rumen microbiota in enteric fermentation and methane production, it is plausible that specific microbial populations play a regulatory role in modulating CH_4_ emissions. The seeding or absence of these population(s) in the rumen environment and intestinal mucosal layer may depend on or modify the expression of *SIGLEC5/14* in the immune system of their host animals. It emphasizes the role of metagenomic analysis aiming to explore the microbiome association with ruminal methane emission in future studies. Emerging evidence indicates that the rumen mucosa is an active immunological interface where host pattern-recognition pathways respond dynamically to changes in the resident microbial community. Several studies have demonstrated that shifts in ruminal microbiota, including methanogenic archaea, are accompanied by alterations in mucosal innate immune gene expression, suggesting that immune–microbiota crosstalk is a key determinant of microbial colonization patterns [41,42,43,44]. There is no current published evidence directly demonstrating *SIGLEC5/14* function in cattle, nor any data connecting bovine *SIGLEC5/14* to the gut- or rumen microbiota. However, SIGLEC family members are expressed and conserved across species, including cattle, indicating functional immune-regulatory roles in this species [45] and, as mentioned earlier, SIGLECs are well-established microbial recognition molecules that detect sialylated microbial, host glycans, and regulate immune responses. Given the extensive two-way interaction between the bovine gut microbiota and host immunity, it is biologically plausible that bovine SIGLECs participate in mediating gut microbe–host interactions [46,47]. Although direct evidence is currently lacking, it is conceivable as a hypothesis that SIGLEC-mediated immune regulation within ruminal mucosal dendritic cells could influence how these cells recognize and respond to methanogenic archaea. If such an interaction exists, it might modulate the extent to which archaea colonize the rumen mucosal surface, potentially functioning as an intermediate phenotype that contributes to animal-level differences in methane emissions. Rumen microbiota were not characterized in the present study, and subsequent shotgun or 16S rRNA sequencing assays are warranted to validate this proposed intermediate phenotype.

On the other hand, KEGG-enriched pathways analysis based on k-means clustering (Figure 6) identified the serotonergic synapse and tryptophan metabolism, serotonin precursor, which is influenced by the gut microbiome. Tryptophan catabolism via kynurenine degradation pathway plays a role in neural activity and systematic inflammatory cascade [48] which adds to the importance of rumen metagenomic studies.

Another immune gene among the DEGs is *IL12B*, which is activated in peripheral blood and mucosal tissues and could plausibly increase the inflammatory tone of the ruminal epithelium, a process known to occur when pro-inflammatory signalling pathways such as NF-κB become upregulated in response to microbial or dietary perturbation [46,49]. Elevated inflammatory tone, partly through *IL12B*, could alter the rumen microenvironment in ways that reduce fibre degradation efficiency probably through suppressing the abundance and activity of key fibrolytic bacterial taxa and the fermentable substrates required for volatile fatty acid (VFA) production, which are central to methanogenesis [50,51].

To enrich potential cell types involved in methane production, PanglaoDB was used in the present study. The PanglaoDB is a web server for probing single-cell RNA sequencing data enrichment of cell types which can help the development of a semi-spatial view, interpretation of gene expression and function and the pathways associations in a tissue-based manner. Our findings reveal that a significant proportion of the enriched cell types predominantly inhabit the epithelial layers of the gastrointestinal tract and the central nervous system. The gut–brain axis concept has emerged to clarify intrinsic and extrinsic factors that influence signalling along this axis. Although more investigation is needed, enrichment of these two groups of genes and cells could be explained, to some extent, based on the findings in this growing field of study [52].

Enriched Enterochromaffin Cells (ECs) are the central players of the gut epithelium that contribute to the protection against potentially harmful contents. More importantly, they respond to the nutrients and microbial secretions, influence metabolism, sense the luminal milieu, and couple to the sensory neural pathways [53,54]. While ECs only comprise 1% of the epithelium, considering the gastrointestinal tract as the largest endocrine organ, these cells significantly participate in endocrine physiology [53]. ECs synthesize more than 30 hormones in mammals, including 95% of secreted serotonin in the body through a calcium influx after the interaction of a ligand, such as a volatile fatty acid, with the olfactory receptor [55,56].

These relations may explain the mechanisms by which the intestine communicates with the nervous system, especially when we consider the enrichment of the Relaxin signalling pathway, as well as the above findings on serotonin (Figure 6). Serotonin is involved in digestive tract motility, secretion, anti-inflammatory activity, and behaviour [57]. The enrichment of the tryptophan metabolism pathway is also in line with this interpretation as it is the only precursor for the neurotransmitter serotonin [48]. Recent advances in exploring the association of digestive- and central nervous systems have clarified the significant contribution of the microbiome in modulating gut–brain signalling and led to the emergence of the microbiota–gut–brain axis [52].

Chondrocytes are the only highly differentiated cells that reside in cartilage [58]. Their apparent enrichment may reflect shared expression of cartilage-related genes, particularly *ACAN*. Crypts are indentations of the epithelium into the underlying mesenchyme, where crypt cells are located. Crypts house the stem cells responsible for the continuous renewal of epithelial cells [59,60]. Stem-cell properties, including expansion potential, self-renewal, and plasticity for differentiation to other cell types, are common among all stem-cell types which necessitate similarity in gene expression pattern among them [61]. Stem-cell properties, including expansion potential, self-renewal, and the plasticity to differentiate into other cell types, are shared across all stem cell types, leading to similarities in their gene expression patterns. 

The enrichment of dendritic cells (DCs) may be attributed to the diverse rumen microbial communities present in the animal’s rumen and intestine that produce varying levels of methane. This variation, in turn, induces the activation of the host immune system in distinct ways, partially through DEGs, among the other required genes. DCs are the most potent and specialized antigen-presenting cells (APCs), expressing both *SIGLEC5* and 14, which play a dual role in the immune system, capable of inducing both activation and tolerance [62,63].

Cell-type enrichment analysis using PanglaoDB revealed signatures associated with blood, gut, and central nervous system cell types. Although the RNA-seq data were derived from whole blood, this observation is biologically plausible given the systemic nature of host–microbiome interactions and the circulation of tissue-derived transcripts via immune cells and extracellular vesicles. The presence of gut- and CNS-associated markers may reflect broader physiological responses to ruminal methane production, consistent with known neuroimmune and gut–immune crosstalk mechanisms [64,65].

Our study identified candidate blood transcriptomic associations with enteric methane in beef-on-dairy cattle. Findings suggest possible involvement of immune and neuroendocrine pathways which need validation in larger datasets and controlled studies. Cautiously, *LOC107131224* (SIGLEC-related) emerges as a candidate for follow-up. The context of NeuroImmunoEndocrinology, as a discipline integrating an intricate system with multidirectional functions and interactions, seems to be the holistic picture that enables the interpretation of these molecular findings [66]. These transcriptomics findings, in line with genomic studies [67,68], emphasize the multi-gene effects of the host genome on methane production. This study, by deciphering the involved host genes, pathways, and mechanisms, may be applied in future studies for the management of methane production. It is notable that the multi-gene nature of ruminal methane production makes it a complex trait, and genetic progress through conventional genetic selection methods will be slow. Therefore, gaining molecular insights of the genetics involved in methane emissions could be leveraged with genomic predictions and rapid genetic progress.

## 5. Conclusions

Within the confines of this small-sample investigation, 11 candidate DEGs (5 upregulated and 6 downregulated) and 9 cell types associated with ruminal methane emissions were identified from paired-end RNA-seq data. Among the genes, the associations of *IL12B* and *KIAA1211L* with methane emissions were relatively robust, and *LOC107131224* is a potential methane-related biomarker that requires further validation in expanded sample cohorts and rumen tissues. These are the preliminary results which need more studies and verification using other data sets; however, they imply that enteric methane production of beef cattle born in dairy herds is influenced by the nervous system, immune system, and endocrine activity of the gastrointestinal tract. We cautiously proposed a conceptual framework, positing ruminal methane production as a NeuroImmunoEndocrinology phenomenon that could open new avenues for understanding the intricate mechanisms underlying methane emissions and help the development of future strategies to identify biomarkers and mitigate GHG production from the livestock industry. One of the limitations in our study was the limited number of animals, hence further investigation is recommended.

## Figures and Tables

**Table 1:** Differentially expressed genes (*p*adj < 0.05).

Gene Symbol	log2 Fold Change	*P*adj	Gene Name	Gene Function
*KIAA1211L*	−0.62	1.22 × 10^−5^	CRACD Like	Miosis, Spermatogenesis, Fertility [26]
*LOC107131224*	−1.28	8.80 × 10^−3^	probably SIGLEC	
*OSCP1*	−0.32	1.21 × 10^−2^	organic solute carrier partner 1	Plasma membrane transporter of organic solutes, Sulfate importer [27,28]
*IL12B*	−0.40	2.68 × 10^−2^	interleukin 12B	Innate and adaptive immunity, Allergic response [29,30]
*LOC618859*	−0.74	1.28 × 10^−2^	Probably interferon omega-1	
*FREM1*	−0.53	2.06 × 10^−2^	FRAS1 related extracellular matrix 1	Extracellular matrix molecule, Cell adhesion [31]
*DSCAML1*	1.62	0.03	DS cell adhesion molecule like 1	Nervous system, Fertility [32]
*OSBP2*	0.88	0.01	oxysterol binding protein 2	Sexual maturation [33]
*ACAN*	1.33	0.01	aggrecan	Skeletal development [34]
*PRSS16*	2.38	0.03	serine protease 16	Immune system, Thymus-specific serine protease [35]
*CD1B*	1.76	0.03	CD1b molecule	Pathogen response, Immune system [36]

**Table 2:** BLAST results for LOC107131224.

mRNA	Accession	Sequence Length	Query Coverage (%)	Percent Identity (%)	E Value
*SIGLEC14*	NM_001098612.3	5134	51	76	7 × 10^−163^
*SIGLEC5* (transcript variant 1)	NM_003830.4	2993	42	76	6 × 10^−129^
*SIGLEC5* (transcript variant 2)	NM_001384708.1	2911	42	76	6 × 10^−129^
*SIGLEC5* (transcript variant 3)	NM_001384709.1	2708	42	76	6 × 10^−129^

**Table 3:** Cell-type enrichment of DEGs.

Term	*p*-Value	Adjusted *p*-Value	Odds Ratio	Genes
Enterochromaffin Cells	0.06	0.12	18.41	*FREM1*
Crypt Cells	0.06	0.12	18.24	*FREM1*
Enteric Glia Cells	0.06	0.12	18.07	*FREM1*
Parietal Cells	0.06	0.12	18.07	*DSCAML1*
Chondrocytes	0.08	0.12	13.69	*ACAN*
Oligodendrocytes	0.09	0.12	11.19	*DSCAML1*
Dendritic Cells	0.1	0.12	10	*CD1B*
Germ Cells	0.12	0.12	8.94	*OSBP2*
Olfactory Epithelial Cells	0.12	0.12	8.78	*OSCP1*

## Data Availability

The original contributions presented in this study are included in the article/Supplementary Material. Further inquiries can be directed to the corresponding author(s).

## Supplementary Materials

The following supporting information can be downloaded at: https://www.mdpi.com/article/10.3390/ani16040589/s1, Figure S1: PCA and UMAP analysis revealed no distinct clustering. Figure S2. Regression analysis indicated no significant effect of breeds on methane measurements; Figure S3. Multiple sequence alignment of LOC107131224 cDNA (transcript: ENSBTAT00000026431.7) and SIGLEC transcripts; Figure S4. Guide tree (A) and Phylogeny tree (B) resulting from multiple sequence alignment; Figure S5. Residual diagnostics for the linear model fitted to LOC107131224 expression; Table S1: Regression analysis indicated no significant effect of breeds on methane measurements; Table S2: Regression analysis revealed that animal age did not significantly affect methane production; Table S3: Regression analysis showed no significant effect of intake on the methane emission; Table S4: KEGG enriched pathways analysis based on k-means clustering; Table S5: GO biological process enriched pathways based on k-means clustering; Table S6: GO molecular function enriched pathways based on k-means clustering; Table S7: GO cellular component enriched pathways based on k-means clustering; Table S8: Core regression results for methane–DEG associations; Table S9: Formal diagnostic tests for model assumptions in DEG–methane regressions; Table S10: Influence-robust and resampling-based validation of DEG–methane slopes; Table S11: Covariate-adjusted methane slopes and Δβ sensitivity analysis; Table S12: Summary of sequencing quality, alignment performance, and read-assignment metrics for all NIFAB-D4B RNA-seq libraries; Table S13: Summary of average PHRED quality scores across representative read positions.

## Abbreviations

The following abbreviations are used in this manuscript:

GHG	Greenhouse Gas
PCA	Principal Component Analysis
UMAP	Uniform Manifold Approximation and Projection
BLAST	Basic Local Alignment Tool
CPM	Counts Per Million
DEGs	Differentially Expressed Genes
SIGLEC	Sialic-acid-binding Immunoglobulin-like Lectin
APC	Antigen Presenting Cell
ECs	Enterochromaffin Cells
HC3	Heteroscedasticity-consistent
LOOCV	Leave-One-Out Cross-Validation
VST	Variance Stabilizing Transformation
DCs	Dendritic Cells
bp	Base Pair
CNS	Central Nervous System
GO	Gene Ontology
KEGG	Kyoto Encyclopedia of Genes and Genomes
FDR	False Discovery Rate
qPCR	Quantitative Polymerase Chain Reaction
CH_4_	Methane
RNA-Seq	RNA Sequencing
MAS	Marker-Assisted Selection
