# Supplementary material for: RNA-Seq Analysis of Ruminal Methane Emissions in Beef-on-Dairy Cattle: Evidence for Immune, Nervous, and Endocrine Pathway Involvement
Source: Animals (Basel). 2026 Feb 13;16(4):589. doi: 10.3390/ani16040589 (PMC12937309; doi:10.3390/ani16040589)
Supplement: Supplementary file 1 [file animals-16-00589-s001.zip › animals-4036711-supplementary.pdf]

**Supporting information:**

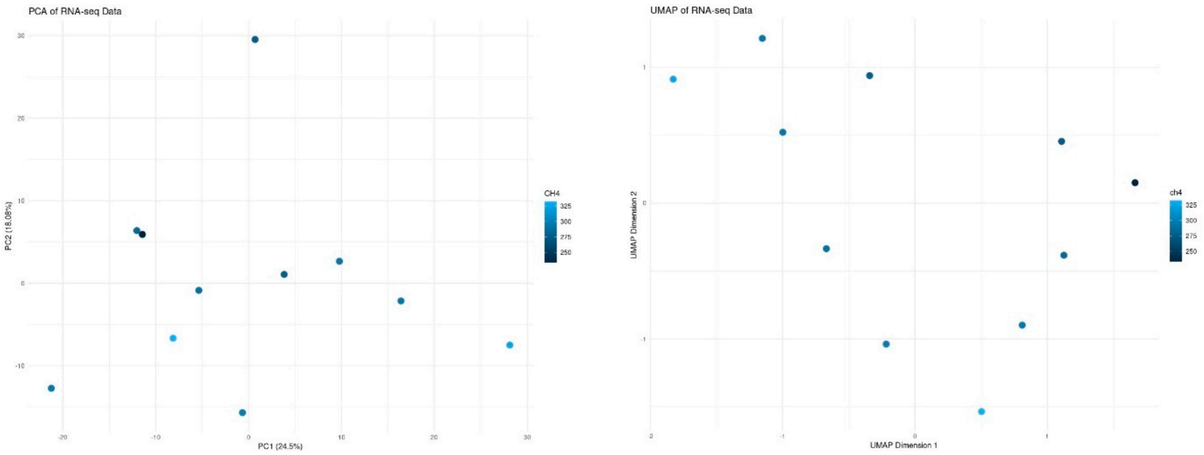


Figure S1. PCA and UMAP analysis revealed no distinct clustering.

ANOVA analysis for animal breeds revealed that the cross-bred animals are not significantly different in methane production (figure 1 and table 1)

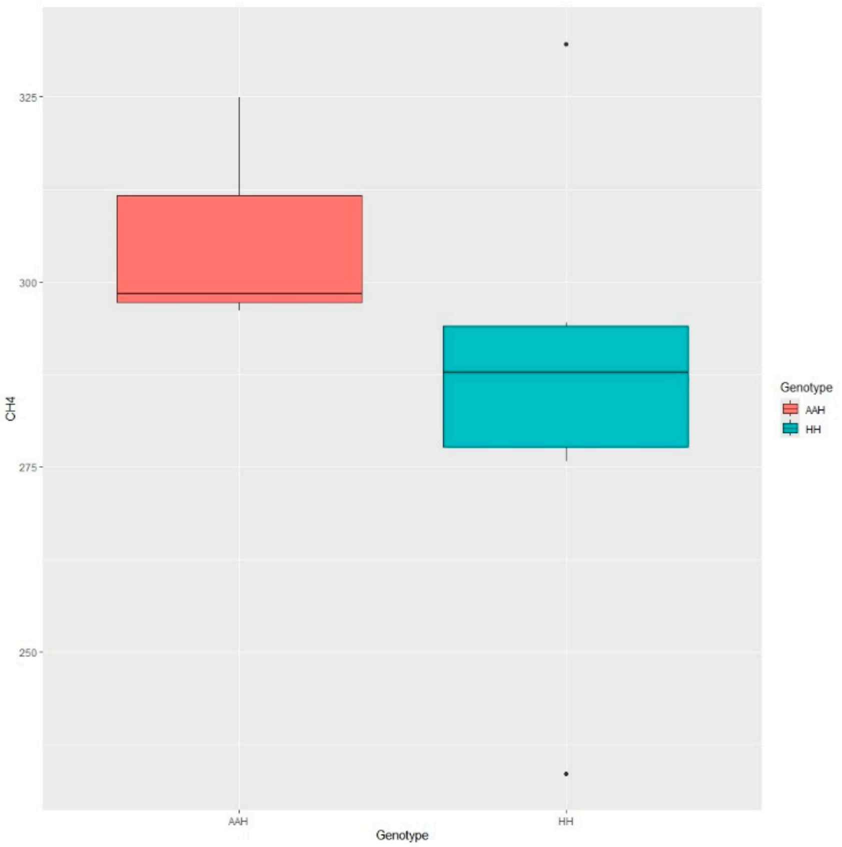


Figure S2. Regression analysis indicated no significant effect of breeds on  methane measurements.

regresion analysis of animal breeds revealed that the crossbred animals were not significantly different in methane production (Figure 1 and Table 1).

Table S1.

| Source | contrast | null.value | estimate | conf.low | conf.high | Adjusted P-value |
|---|---|---|---|---|---|---|
| Genotype | HH-AAH | 0 | -21.0342 | -59.63 | 17.56158 | 0.248864 |

The ages for the animals were calculated using lubridate R package. The ages renaged from 1.57 to 1.97 years, corresponding to 574 to 721 days, at the beginning of the study.  The regression analysis revealed that animal age did not significantly affect methane production (table 2)

Table S2.

| Source | df | SS | MS | F | P-value |
|---|---|---|---|---|---|
| Age | 1 | 130.8245 | 130.8245 | 0.179744 | 0.681547 |
| Residuals | 9 | 6550.542 | 727.838 | NA | NA |

The average feed intake of each animal was measured for 35 days during the experiment, and the regression analysis showed no significant effect of intake on the methane emission (table 3).

Table S3.

| Source | df | SS | MS | F | P-value |
|---|---|---|---|---|---|
| Feed intake | 1 | 1653.498 | 1653.498 | 2.9598 | 0.119469 |
| Residuals | 9 | 5027.868 | 558.652 | NA | NA |

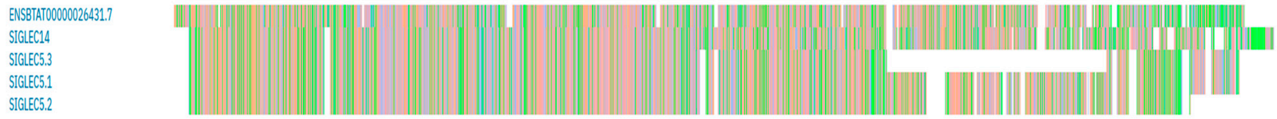


Figure S3. Multiple sequence alignment of LOC107131224 cDNA (transcript: ENSBTAT00000026431.7) and SIGLEC transcripts

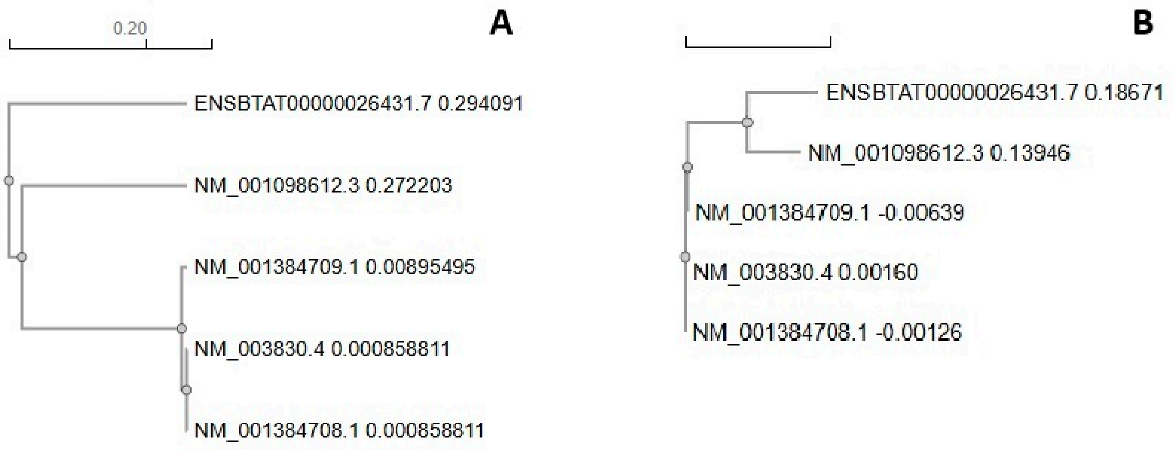


Figure S4. Guide tree (A) and Phylogeny tree (B) resulting from multiple sequence alignment.

Table S4. KEGG enriched pathways analysis based on k-means clustering

| Cluster # | FDR | nGenes | Pathway size | Fold enriched | Pathway |
|---|---|---|---|---|---|
| 1 | 0.0005 | 14 | 88 | 4.37 | ECM-receptor interaction |
| 1 | 0.0005 | 14 | 103 | 4.24 | Protein digestion and absorption |
| 1 | 0.08 | 7 | 61 | 4.00 | Arachidonic acid metabolism |
| 1 | 0.08 | 19 | 200 | 2.19 | Focal adhesion |
| 1 | 0.08 | 7 | 94 | 4.1130512 | Staphylococcus aureus infection |
| 1 | 0.09 | 25 | 354 | 1.83 | PI3K-Akt signaling pathway |
| 1 | 0.09 | 25 | 331 | 1.83 | Human papillomavirus infection |
| 2 | 0.001 | 24 | 362 | 2.67 | Neuroactive ligand-receptor interaction |
| 2 | 0.001 | 14 | 86 | 3.97 | Insulin secretion |
| 2 | 0.004 | 9 | 61 | 4.80 | Arachidonic acid metabolism |
| 2 | 0.004 | 17 | 129 | 2.90 | Relaxin signaling pathway |
| 2 | 0.004 | 13 | 102 | 3.62 | Pancreatic secretion |
| 2 | 0.02 | 13 | 114 | 3.00 | Glutamatergic synapse |
| 2 | 0.02 | 8 | 42 | 4.14 | Tryptophan metabolism |
| 2 | 0.02 | 11 | 103 | 3.11 | Protein digestion and absorption |
| 2 | 0.06 | 20 | 294 | 2.03 | Cytokine-cytokine receptor interaction |
| 2 | 0.07 | 11 | 112 | 2.70 | Serotonergic synapse |

Table S5. GO biological process enriched pathways based on k-means clustering

| Group | FDR | nGenes | Pathway size | Fold enriched | Pathway |
|---|---|---|---|---|---|
| 1 | 9.76×10^-12 | 84 | 943 | 2.49 | Cell adhesion |
| 1 | 9.76×10^-12 | 84 | 949 | 2.48 | Biological adhesion |
| 1 | 7.86×10^-8 | 216 | 3856 | 1.49 | Cellular developmental process |
| 1 | 9.06×10^-8 | 212 | 3790 | 1.49 | Cell differentiation |
| 1 | 1.31×10^-7 | 236 | 4336 | 1.43 | System development |
| 1 | 1.34×10^-7 | 20 | 142 | 5.54 | Homophilic cell adhesion via plasma membrane adhesion molecules |
| 1 | 3.47×10^-6 | 131 | 2164 | 1.63 | Anatomical structure morphogenesis |
| 1 | 1.59×10^-5 | 39 | 444 | 2.63 | Cell-cell adhesion |
| 1 | 1.96×10^-5 | 24 | 237 | 3.60 | Cell-cell adhesion via plasma-membrane adhesion molecules |
| 1 | 2.77×10^-5 | 75 | 986 | 1.90 | Regulation of cellular component movement |
| 2 | 2.53×10^-8 | 69 | 1816 | 2.42 | G protein-coupled receptor signaling pathway |
| 2 | 1.03×10^-6 | 106 | 1630 | 1.81 | Ion transport |
| 2 | 1.03×10^-6 | 80 | 1074 | 2.02 | Cation transport |
| 2 | 1.03×10^-6 | 63 | 756 | 2.27 | Metal ion transport |
| 2 | 1.20×10^-6 | 50 | 565 | 2.51 | Monovalent inorganic cation transport |
| 2 | 1.20×10^-6 | 63 | 764 | 2.23 | Inorganic cation transmembrane transport |
| 2 | 1.54×10^-6 | 26 | 283 | 3.85 | Defense response to bacterium |
| 2 | 1.88×10^-6 | 85 | 1243 | 1.92 | Ion transmembrane transport |
| 2 | 2.23×10^-6 | 67 | 877 | 2.11 | Inorganic ion transmembrane transport |

Table S6. GO molecular function enriched pathways based on k-means clustering

| Group | FDR | nGenes | Pathway size | Fold enriched | Pathway |
|---|---|---|---|---|---|
| 1 | 7.69×10^-9 | 20 | 95 | 6.41 | Extracellular matrix structural constituent |
| 1 | 4.45×10^-8 | 71 | 849 | 2.231 | Calcium ion binding |
| 1 | 1.07×10^-6 | 28 | 265 | 3.561 | Glycosaminoglycan binding |
| 1 | 7.77×10^-6 | 34 | 295 | 2.84 | Cell adhesion molecule binding |
| 1 | 5.29×10^-5 | 21 | 208 | 3.55 | Heparin binding |
| 1 | 5.29×10^-5 | 44 | 720 | 2.27 | Receptor regulator activity |
| 1 | 2.08×10^-4 | 99 | 1789 | 1.59 | Signaling receptor binding |
| 1 | 2.08×10^-4 | 23 | 217 | 3.03 | Iron ion binding |
| 1 | 3.15×10^-4 | 74 | 2135 | 1.70 | Signaling receptor activity |
| 1 | 3.15×10^-4 | 40 | 688 | 2.17 | Receptor ligand activity |
| 2 | 1.11×10^-11 | 81 | 1887 | 2.43 | Transmembrane signaling receptor activity |
| 2 | 1.11×10^-11 | 95 | 2135 | 2.24 | Signaling receptor activity |
| 2 | 1.11×10^-11 | 95 | 2135 | 2.24 | Molecular transducer activity |
| 2 | 4.63×10^-8 | 48 | 498 | 2.72 | Metal ion transmembrane transporter activity |
| 2 | 5.86×10^-8 | 61 | 730 | 2.35 | Inorganic cation transmembrane transporter activity |
| 2 | 1.03×10^-6 | 78 | 1121 | 1.95 | Ion transmembrane transporter activity |
| 2 | 1.08×10^-6 | 45 | 1470 | 2.53 | G protein-coupled receptor activity |

| | | | | | |
|---|---|---|---|---|---|
| 2 | 1.14×10^-6 | 61 | 792 | 2.14 | Cation transmembrane transporter activity |
| 2 | 1.14×10^-6 | 73 | 1037 | 1.98 | Inorganic molecular entity transmembrane transporter activity |

Table S7. GO cellular component enriched pathways based on k-means clustering

| Group | FDR | nGenes | Pathway size | Fold enriched | Pathway |
|---|---|---|---|---|---|
| 1 | 3.54×10^-40 | 214 | 3098 | 2.51 | Extracellular region |
| 1 | 4.55×10^-18 | 119 | 1845 | 2.40 | Extracellular space |
| 1 | 1.49×10^-15 | 54 | 436 | 3.77 | Extracellular matrix |
| 1 | 1.77×10^-12 | 99 | 1357 | 2.21 | Integral component of plasma membrane |
| 1 | 2.82×10^-12 | 102 | 1428 | 2.16 | Intrinsic component of plasma membrane |
| 1 | 7.22×10^-11 | 35 | 259 | 4.04 | Collagen-containing extracellular matrix |
| 1 | 2.06×10^-6 | 59 | 771 | 2.12 | Cell surface |
| 1 | 4.46×10^-6 | 87 | 1355 | 1.77 | Plasma membrane region |
| 1 | 6.72×10^-6 | 15 | 119 | 5.13 | Collagen trimer |
| 1 | 5.18×10^-5 | 14 | 77 | 4.70 | Presynaptic active zone |
| 2 | 1.43×10^-21 | 175 | 3098 | 2.10 | Extracellular region |
| 2 | 3.06×10^-18 | 114 | 1428 | 2.47 | Intrinsic component of plasma membrane |
| 2 | 7.21×10^-16 | 105 | 1357 | 2.41 | Integral component of plasma membrane |
| 2 | 4.99×10^-15 | 110 | 1845 | 2.28 | Extracellular space |
| 2 | 1.53×10^-7 | 40 | 436 | 2.87 | Extracellular matrix |
| 2 | 1.59×10^-6 | 25 | 224 | 3.63 | Cation channel complex |

| | | | | | |
|---|---|---|---|---|---|
| 2 | 1.93×10^-6 | 31 | 346 | 3.05 | Transmembrane transporter complex |
| 2 | 3.77×10^-6 | 8 | 25 | 11.87 | Complex of collagen trimers |
| 2 | 4.56×10^-6 | 48 | 681 | 2.26 | Plasma membrane protein complex |
| 2 | 4.56×10^-6 | 31 | 357 | 2.90 | Transporter complex |

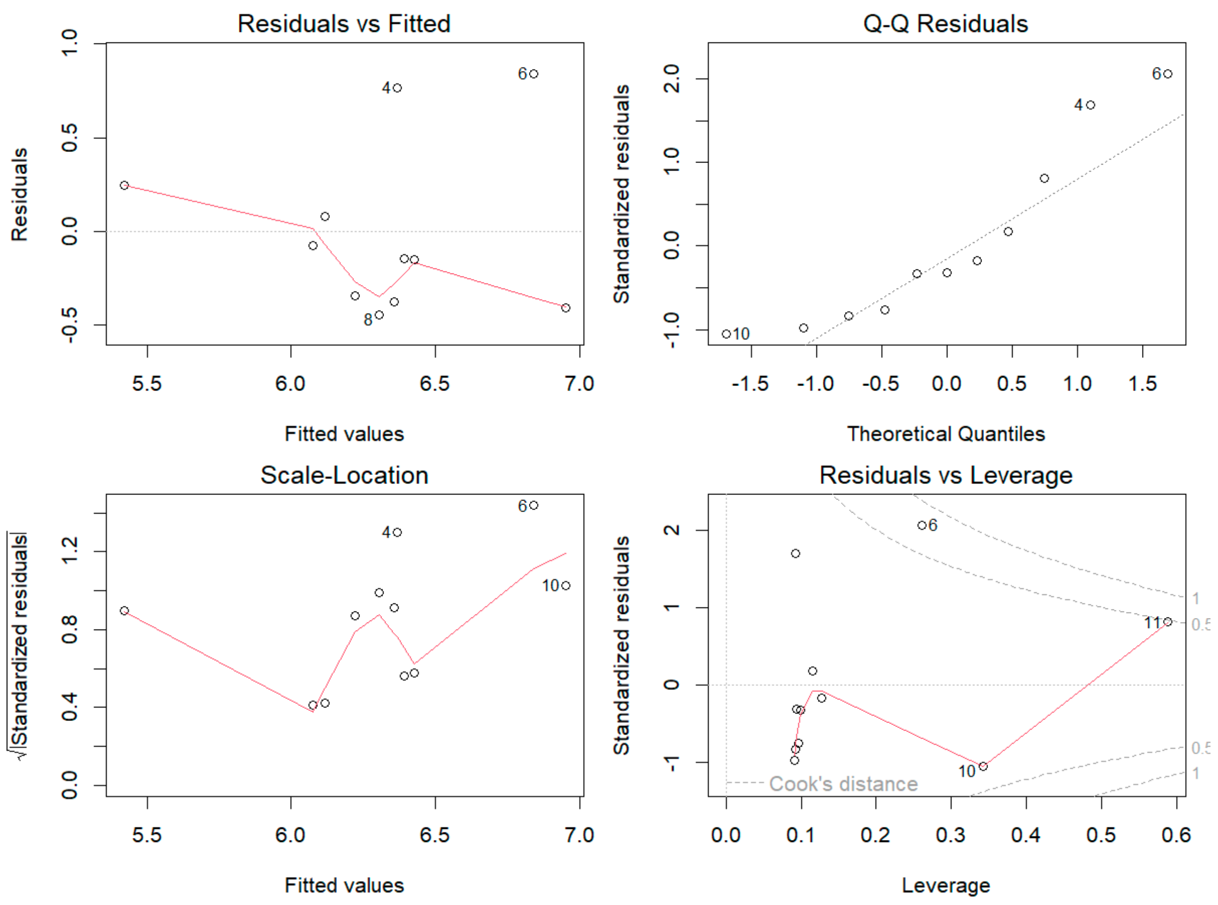


Figure S5. Residual diagnostics for the linear model fitted to LOC107131224 expression. Residuals vs Fitted: Residuals show a largely symmetric distribution around zero with only mild curvature in the LOESS trend, indicating no substantial violations of linearity or mean–variance structure. Normal Q–Q plot: Most standardized residuals follow the theoretical normal line, with moderate deviation in the upper tail driven by a small number of animals. Scale–Location plot: The spread of √|standardized residuals| remains relatively stable across fitted values, suggesting only minor heteroscedasticity.

Residuals vs Leverage: No observations exceed Cook's distance thresholds, indicating that no single animal exerted undue influence on model estimates.

Table S8. Core regression results for methane–DEG associations.

For each DEG, a simple linear model (expr ~ methane) was fitted to VST-normalized expression values. Reported are the ordinary least squares (OLS) slope (β) for methane, its 95% confidence interval, the associated p-value, and the adjusted $R^2$. These results summarize the primary strength and direction of each methane–expression relationship prior to any robustness or assumption testing.

| Gene | β (methane) | 95% CI (β) | P (OLS) | Adj R2 |
|---|---|---|---|---|
| KIAA1211L | -0.01808 | [-0.02489,-0.01127] | 0.000202 | 0.778 |
| LOC107131224 | -0.03940 | [-0.06319,-0.01562] | 0.00457 | 0.566 |
| OSCP1 | -0.00793 | [-0.01089,-0.00497] | 0.000188 | 0.781 |
| IL12B | -0.00979 | [-0.01408,-0.00550] | 0.000595 | 0.719 |
| LOC618859 | -0.01014 | [-0.01429,-0.00599] | 0.000365 | 0.747 |
| FREM1 | -0.00846 | [-0.01194,-0.00495] | 0.000394 | 0.743 |
| DSCAML1 | 0.01512 | [0.00238,0.02786] | 0.0250 | 0.383 |
| OSBP2 | 0.01748 | [0.00558,0.02937] | 0.00890 | 0.501 |
| ACAN | 0.01625 | [0.00738,0.02513] | 0.00251 | 0.618 |
| PRSS16 | 0.01275 | [0.00354, 0.02196] | 0.0121 | 0.468 |
| CD1B | 0.01825 | [0.00380, 0.03270] | 0.0189 | 0.417 |

Table S9. Formal diagnostic tests for model assumptions in DEG–methane regressions.

Assumption checks include p-values for the Shapiro–Wilk test of residual normality, the Breusch–Pagan (BP) test for homoscedasticity, and the Ramsey RESET test for model specification. Influence diagnostics identify samples flagged by Cook's distance > 4/n or |studentized residual| > 2. These results complement the visual residual diagnostics and confirm whether each fitted model meets standard linear regression assumptions.

| Gene | Shapiro | BP | RESET | Influential |
|---|---|---|---|---|
| KIAA1211L | 0.6738 | 0.7690 | 0.0132 | Cook:10; stud:2 |
| LOC107131224 | 0.3734 | 0.3196 | 0.0390 | Cook:11; stud:5,11 |
| OSCP1 | 0.4250 | 0.4950 | 0.0316 | Cook:6,11; stud:11 |
| IL12B | 0.4152 | 0.0484 | 0.2726 | Cook:6,10,11; stud:6,10 |

| | | | | |
|---|---|---|---|---|
| LOC618859 | 0.2844 | 0.1419 | 0.4602 | Cook:10,11; stud:10 |
| FREM1 | 0.3594 | 0.3495 | 0.133 | Cook:10,11; stud:— |
| DSCAML1 | 0.0239 | 0.1115 | 0.5563 | Cook:6,11; stud:6 |
| OSBP2 | 0.9427 | 0.1819 | 0.1441 | Cook:10,11; stud:10 |
| ACAN | 0.3965 | 0.7002 | 0.4996 | Cook:11; stud:7 |
| PRSS16 | 0.1818 | 0.4348 | 0.2869 | Cook:6,11; stud:6 |
| CD1B | 0.0430 | 0.1250 | 0.6266 | Cook:6,11; stud:6 |

Table S10. Influence-robust and resampling-based validation of DEG–methane slopes.

Robustness was evaluated using: leave-one-out cross-validation (LOOCV) to assess how omission of any single animal affects the slope estimate; heteroscedasticity-consistent HC3 p-values to guard against variance misspecification; and non-parametric pairs bootstrap 95% confidence intervals based on 2,000 resamples. Together, these analyses provide a stability profile for each DEG, accounting for small-sample uncertainty and mild violations of regression assumptions.

| Gene | LOOCV Range | HC3 p | Bootstrap CI |
|---|---|---|---|
| KIAA1211L | [-0.02075,-0.01701] | 0.000513 | [-0.0367,-0.0130] |
| LOC107131224 | [-0.06271,-0.03524] | 0.1357 | [-0.0902,-0.0211] |
| OSCP1 | [-0.00898,-0.00461] | 0.0516 | [-0.0114,-0.0028] |
| IL12B | [-0.01186,-0.00746] | 0.0243 | [-0.0191,-0.0065] |
| LOC618859 | [-0.01233,-0.00814] | 0.0104 | [-0.0138,-0.0043] |
| FREM1 | [-0.00975,-0.00562] | 0.0265 | [-0.0123,-0.0029] |
| DSCAML1 | [0.00871,0.02143] | 0.1537 | [0.0059,0.0383] |
| OSBP2 | [0.00939,0.02526] | 0.163 | [-0.0022,0.0296] |
| ACAN | [0.01511,0.02004] | 0.00288 | [0.0125,0.0291] |
| PRSS16 | [0.00860,0.01972] | 0.1532 | [0.0045,0.0296] |
| CD1B | [0.00977,0.02070] | 0.1054 | [0.0074,0.0375] |

Table S11. Covariate-adjusted methane slopes and Δβ sensitivity analysis.

For each DEG, unadjusted models (expr ~ methane) were compared with covariate-adjusted models including age, feed intake, and breed. Covariates were screened via variance-inflation factors (VIF), and any variable with VIF ≥ 5 was excluded to avoid multicollinearity. Δβ = β_adjusted − β_unadjusted, where positive values indicate slope attenuation and negative values indicate slope strengthening after adjustment. These results quantify potential bias due to sample homogeneity and evaluate whether animal-level characteristics influence the methane–expression associations.

| Gene | β_unadj | 95%CI | p_unadj | AdjR2 | β_adj | Δβ |
|---|---|---|---|---|---|---|
| KIAA1211L | -0.018077 | [-0.024888,-0.011265] | 0.000202 | 0.777983 | -0.014956 | 0.003120 |
| LOC107131224 | -0.039405 | [-0.063189,-0.015620] | 0.004570 | 0.566083 | -0.034088 | 0.005317 |
| OSCP1 | -0.007930 | [-0.010891,-0.004970] | 0.000188 | 0.781252 | -0.008341 | -0.000411 |
| IL12B | -0.009792 | [-0.014084,-0.005500] | 0.000595 | 0.719351 | -0.007795 | 0.001997 |
| LOC618859 | -0.010144 | [-0.014292,-0.005995] | 0.000365 | 0.747415 | -0.010377 | -0.000233 |
| FREM1 | -0.008446 | [-0.011938,-0.004954] | 0.000394 | 0.743182 | -0.009957 | -0.001511 |
| DSCAML1 | 0.015120 | [0.002376,0.027863] | 0.025044 | 0.382849 | 0.014298 | -0.000822 |
| OSBP2 | 0.017475 | [0.005579,0.029371] | 0.008896 | 0.501071 | 0.018516 | 0.001041 |
| ACAN | 0.016253 | [0.007379,0.025126] | 0.002509 | 0.617857 | 0.015473 | -0.000779 |
| PRSS16 | 0.012750 | [0.003541,0.021959] | 0.012082 | 0.468357 | 0.010798 | -0.001953 |
| CD1B | 0.018250 | [0.003796,0.032703] | 0.018895 | 0.417193 | 0.016290 | -0.001959 |

Table S12. Summary of sequencing quality, alignment performance, and read-assignment metrics for all NIFAB-D4B RNA-seq libraries

| ID | M Seqs | % GC | % Dups | % Failed | % Aligned | M Aligned | % Assigned | M Assigned |
|---|---|---|---|---|---|---|---|---|
| 36206 | 44 | 48 | 38.4 | 18 | 68.5 | 29.3 | 35.8 | 10.5 |
| 36206 | 44 | 48 | 38 | 27 | 68.5 | 29.3 | 35.8 | 10.5 |
| 36556 | 52.8 | 48 | 37.2 | 18 | 71.2 | 36.7 | 32.3 | 11.8 |
| 36556 | 52.8 | 48 | 36.7 | 27 | 71.2 | 36.7 | 32.3 | 11.8 |
| 36851 | 53.7 | 49 | 37.9 | 18 | 69.7 | 36.5 | 32 | 11.7 |
| 36851 | 53.7 | 49 | 37.6 | 27 | 69.7 | 36.5 | 32 | 11.7 |
| 37046 | 53.1 | 49 | 37.7 | 18 | 68.9 | 35.6 | 32.3 | 11.5 |
| 37046 | 53.1 | 49 | 37.1 | 27 | 68.9 | 35.6 | 32.3 | 11.5 |
| 37201 | 45.5 | 49 | 38.9 | 27 | 67.7 | 30 | 34.6 | 10.4 |
| 37201 | 45.5 | 49 | 38.6 | 27 | 67.7 | 30 | 34.6 | 10.4 |
| 37223 | 47.8 | 50 | 38.4 | 18 | 64.9 | 30.3 | 35.7 | 10.8 |
| 37223 | 47.8 | 51 | 38.2 | 27 | 64.9 | 30.3 | 35.7 | 10.8 |
| 37267 | 51.7 | 48 | 36.5 | 18 | 70.1 | 35.6 | 32 | 11.4 |
| 37267 | 51.7 | 48 | 36.6 | 18 | 70.1 | 35.6 | 32 | 11.4 |
| 37282 | 48.6 | 47 | 37.6 | 18 | 67.4 | 32.1 | 31.2 | 10 |
| 37282 | 48.6 | 48 | 37.3 | 27 | 67.4 | 32.1 | 31.2 | 10 |
| 37304 | 42.1 | 48 | 36.3 | 18 | 67.8 | 27.9 | 32.7 | 9.1 |
| 37304 | 42.1 | 48 | 35.8 | 18 | 67.8 | 27.9 | 32.7 | 9.1 |
| 37326 | 56.2 | 48 | 37.5 | 18 | 69.7 | 38.2 | 31.4 | 12 |
| 37326 | 56.2 | 49 | 37.3 | 27 | 69.7 | 38.2 | 31.4 | 12 |
| 38096 | 55.1 | 48 | 38.1 | 18 | 70.5 | 37.9 | 32.5 | 12.3 |
| 38096 | 55.1 | 48 | 37.7 | 36 | 70.5 | 37.9 | 32.5 | 12.3 |
| 38111 | 60.2 | 48 | 39.1 | 18 | 70.4 | 41.4 | 31.4 | 13 |
| 38111 | 60.2 | 48 | 38.5 | 27 | 70.4 | 41.4 | 31.4 | 13 |

Table S13. Summary of average PHRED quality scores across representative read positions. All positions exhibit PHRED scores ≥34, corresponding to base-calling accuracies of ≥99.96%, indicating excellent sequencing quality throughout the read length.

| Read Position (bp) | Average Phred Score (Q) | Estimated Accuracy |
|---|---|---|
| 1 | 34 | 99.96% |
| 10 | 37 | 99.98% |
| 20 - 40 | 38 | 99.98% |
| 60 | 37 | 99.98% |
| 80 | 36 | 99.97% |
| 100+ | 35 | 99.97% |
